# Evaluation of oral cholera vaccine (Euvichol-Plus) effectiveness against Vibrio cholerae in Bangladesh: an interim analysis

**DOI:** 10.1136/bmjgh-2024-016571

**Published:** 2025-02-03

**Authors:** Farhana Khanam, Md. Taufiqul Islam, Faisal Ahmmed, Md Nazmul Hasan Rajib, Md Ismail Hossen, Fahima Chowdhury, Ashraful Islam Khan, Md. Taufiqur Rahman Bhuiyan, Shahinur Haque, Prasanta Kumar Biswas, Amirul Islam Bhuiyan, Zahid Hasan Khan, Mohammad Ashraful Amin, Aninda Rahman, S M Shahriar Rizvi, Tahmina Shirin, Md Nazmul Islam, Amanda Tiffany, Lucy Breakwell, Firdausi Qadri, John D Clemens

**Affiliations:** 1 Infectious Diseases Division, International Centre for Diarrhoeal Disease Research Bangladesh, Dhaka, Dhaka District, Bangladesh; 2 Communicable Disease Control, Dhaka, Bangladesh; 3 Institute of Epidemiology Disease Control and Research, Dhaka, Dhaka District, Bangladesh; 4 Global Immunization Division, Centers for Disease Control and Prevention, Atlanta, Georgia, USA; 5 International Vaccine Institute, Gwanak-gu, Seoul, Korea (the Republic of); 6 UCLA Fielding School of Public Health, Los Angeles, California, USA

**Keywords:** Global Health, Vaccines, Cholera

## Abstract

**Introduction:**

Millions of Euvichol-Plus doses have been deployed from the global oral cholera vaccine stockpile in over 20 cholera-affected countries. However, information on Euvichol-Plus’s effectiveness is limited. Using this vaccine in a cholera epidemic in Dhaka, Bangladesh, provided the opportunity to evaluate the vaccine effectiveness (VE) using a test-negative design.

**Methods:**

A two-dose regimen of Euvichol-Plus was administered to individuals aged >1 year in a population of ca. 900 000 in two campaign rounds between June and August 2022, with prospective registration of all persons who received at least one dose. We conducted systematic surveillance in two key facilities, enrolling patients with acute watery diarrhoea who were eligible for vaccination from the campaign’s start and who presented for care between 21 August 2022 and 20 August 2023. Faecal culture-positive cholera cases were matched to up to four faecal culture-negative controls by age, presentation date and facility. Vaccination status was documented without knowledge of culture results. Conditional logistic regression models estimated the OR for the vaccination-cholera association, and the VE of the two-dose regimen was calculated as [(1−OR) × 100].

**Results:**

The analysis included 226 cases and 552 matched controls. The adjusted VE of two doses of the Euvichol-Plus vaccine against medically attended cholera was 66% (99.5% CI: 30 to 83) for all recipients. Limited protection (12%; 95% CI: −95 to 60) was observed for children aged 1–4 years; whereas, protection was 79% (95% CI: 60 to 89) for those aged ≥5 years. VE against cholera with moderate to severe dehydration was 69% (95% CI: 44 to 83) overall but 6% (95% CI: −206 to 71) for children aged 1–4 years.

**Conclusion:**

Euvichol-Plus provided significant protection against medically attended cholera of any severity as well as cholera with moderate to severe dehydration. However, significant levels of protection were only observed for those aged ≥5 years.

**WHAT IS ALREADY KNOWN ON THIS TOPIC**

**WHAT THIS STUDY ADDS**

- A large-scale roll-out of Euvichol-Plus in Dhaka, Bangladesh, in response to a major cholera outbreak offered the opportunity to conduct the effectiveness study using a test-negative design.

**HOW THIS STUDY MIGHT AFFECT RESEARCH, PRACTICE OR POLICY**

- The study demonstrated significant protection against cholera for individuals aged ≥5 years provided by the Euvichol-Plus vaccine. Given that children under five represent a large portion of the cholera burden, the limited protection observed in this age group underscores the need for ongoing research to improve the effectiveness of Euvichol-Plus.

## Introduction

 Cholera outbreaks cause high morbidity and mortality across the world.^1^ The WHO recommends the use of oral cholera vaccines (OCVs) together with traditional control measures, including the provision of safe water, and improved hygiene and sanitation, in cholera-endemic areas and during cholera outbreaks.^2^ Since 2013, Gavi has supported the global OCV stockpile, coordinated by WHO and other international organisations, to facilitate access to WHO prequalified OCVs.^3^ Shanchol (produced by Shantha Biotechnics Ltd., India and WHO prequalified in 2011), a bivalent, O1–O139 serogroup inactivated whole-cell vaccine, was the first WHO prequalified vaccine to be included in the OCV stockpile but is no longer manufactured.^4 5^ Euvichol (produced by EuBiologics Co., South Korea and WHO prequalified in 2015), a vaccine identical to Shanchol and produced by a different manufacturer, was later added to the OCV stockpile in the same year,^6^ followed by Euvichol-Plus (produced by EuBiologics Co., South Korea and WHO prequalified in 2017), identical in composition to Euvichol but presented in single-dose, flexible plastic tubes that allow for convenient, direct vaccine administration, in contrast to Shanchol and Euvichol which are injectable vaccines that are stored in glass vials. Euvichol-Plus was added to the stockpile in 2017, approved by the WHO.^7^

Although millions of doses of Euvichol-Plus have been dispensed from the OCV stockpile, only two studies conducted in Zambia and the Democratic Republic of the Congo (DRC) have evaluated its protective effectiveness. In Zambia, a case-control study showed that within 6 months of vaccination, the protective effectiveness of two doses was 81% and at least one dose was 74%, among individuals aged 1 year and older. However, the study did not find evidence of protection in children aged 1–4 years at presentation for cholera.^8^ A case-control study in the DRC reported that protection from a single dose of Euvichol-Plus was 52.7% at 12–17 months and 44.7% from a single dose of a two-dose regimen of the vaccine during 12–17 months and 24–36 months after vaccination.^9^

In March 2022, Dhaka experienced an unusually large outbreak of cholera. In response to this outbreak, the government of Bangladesh (GOB) implemented a large-scale reactive OCV campaign with a two-dose regimen of Euvichol-Plus. We conducted a prospective evaluation of the protection conferred by a complete two-dose regimen in two high-risk vaccinated areas of Dhaka city using a test-negative design and reported our findings after 1 year of follow-up.

## Methods

### Description of the epidemic

Cholera is known to have biannual seasonal peaks in Dhaka city, Bangladesh, one between April and May and the second between August and September, with the highest annual peak usually seen around April. In 2022, a major cholera surge began in early March, with approximately 600 patients per day seeking care for acute watery diarrhoea (AWD) at the icddr,b Dhaka Hospital. The number of patients continued to increase with around 1300 new patients treated each day by the second week of March. The proportion of culture-confirmed cholera among all diarrhoeal patients treated at the hospital increased from 11% in early March to 34% by 23 March, with a peak of 36% in the second week of April 2022. More than 50% of the hospitalised patients with diarrhoea came from Jatrabari, Sabujbagh, Dakshinkhan, Mohammadpur and Mirpur thanas (an administrative unit of the district) in Dhaka city, which were considered cholera hotspots or high-risk areas. At the same time, the number of patients seeking care for diarrhoea also increased in other government hospitals in the city.

### Vaccination campaign in cholera Hotspots

The Communicable Disease Control Programme of the Directorate General of Health Services of the GOB, in collaboration with the icddr,b, and other partners, implemented a reactive OCV campaign for non-pregnant individuals aged 1 year and above with a two-dose regimen of Euvichol-Plus. Five thanas in Dhaka city, identified as cholera hotspots due to the number of suspected cases reported from those areas, with a combined population of approximately 2.4 million residents, were prioritised for vaccination. GOB received 4.75 million vaccines from the global stockpile for the two-dose campaign on 11 April 2022.^10^ After a delay due to the fasting season of Ramadan, and based on EPI (Expanded Programme on Immunisation) and MoHFW (Ministry of Health and Family Welfare) suitability of time, two rounds of vaccination were carried out: the first from 26 June to 2 July 2022, and the second from 3 August to 10 August 2022. We evaluated the protective effectiveness of Euvichol-Plus in two of the thanas, Mirpur and Dakshinkhan, where approximately 900 000 eligible individuals were vaccinated during the campaign. A structured form was used to collect names, permanent and present addresses, mobile number, and number and dates of doses received for each vaccinee in the study areas during the vaccination campaign. These data were entered into an electronic database and used to construct a vaccination database.

### Diarrhoeal disease surveillance and sampling frame for the test-negative study

Individuals in two of the thanas, Mirpur and Dakshinkhan, who were residents at the onset of the vaccination campaign, were followed as a closed cohort for the study. Facility-based disease surveillance was initiated on 4 July 2022, in the icddr,b Dhaka Hospital, and Dakshinkhan Field Office, two major sources of care for diarrhoea for residents of the study areas. Patients with AWD attending the outpatient or inpatient units of the designated health facilities were approached for enrollment. AWD was defined as any patient attending the hospital with 3 or more loose or liquid stools within 24 hours or less than 3 loose/liquid stools causing dehydration. Patients aged 1 year or older on the first day of the vaccination campaign who had resided in the study area since the initiation of the campaign and presented with a history of acute watery diarrhoea of ≤7 days duration without a history of diarrhoea during the 7 days before the onset of the current illness were considered eligible and approached for enrollment. Patient enrollment was carried out in each designated health facility from 08:30 to 17:00 hours, 6 days a week, and written informed consent was obtained from each participant. Study nurses verified the patients’ eligibility at the time of enrollment. A study physician collected demographic, socioeconomic and clinical data, including history and physical examination, through a structured questionnaire. The patient’s dehydration status at the time of presentation was assessed using WHO dehydration assessment criteria.^11 12^

### Specimen collection and laboratory testing

Stool or rectal swab specimens were collected from all enrolled participants and transported to the icddr,b laboratory while maintaining a temperature of 2–8°C. To detect *Vibrio cholerae*, specimens were enriched in alkaline peptone water overnight and then cultured on selective taurocholate-tellurite gelatine agar media.^13^ Specific monoclonal antibodies were used to determine the serotypes (Ogawa and Inaba) of *V. cholerae* O1, and bio-typing of strains was also carried out.^14^ Culture negative stool samples were also tested by PCR of the *V. cholerae* specific rfb gene. However, PCR results were not included as the interim analysis focused on the primary objectives of the protocol.

### Definitions and assembly of test-positive cases and test-negative controls

An otherwise eligible diarrhoeal patient positive by stool culture for *V. cholerae* was considered a test-positive case, and an otherwise eligible diarrhoeal patient who was negative by stool culture for *V. cholerae* was considered a test-negative control. Focal time was defined as the date of the onset of diarrhoea for both cases and controls and used to calculate age for the purpose of matching cases and controls in addition to assessing time between vaccination and disease onset. Both the ascertainment of eligibility and the collection of clinical, demographic and socioeconomic data were done in a manner blinded to the patient’s status as a test-positive case or a test-negative control. A statistician, blinded to the vaccination status of participants, ordered eligible cholera cases according to their focal times in calendar time, starting from the inception of surveillance, and for each sequential case randomly selected up to four matched culture-negative controls. Cases and controls were matched on three characteristics: age at focal time (1–4 years; ≥5–17 years; 18–60 years; 60+ years), date of presentation and health facility. Controls were randomly selected on the day of the presentation of the case; if four matched controls were not available on that day, the sampling interval was progressively widened for up to a maximum of ±7 days, successively inspecting potential controls 1 day before and then 1 day after the date of case selection. Cases and controls were selected using incidence density sampling: a case could not later be re-selected as a case or a control, but a control could later be re-selected as a control or a case.

### Acquisition of data on vaccination status

During enrollment, a trained study staff member ascertained receipt of OCV from all eligible patients who consented to participation. If the patient presented a study vaccine card giving receipt details, this was accepted. If the patient failed to bring the card, the vaccination database created for the study was checked. All information about vaccination, including number of doses received, was obtained by study staff who were blinded to the faecal culture result.

### Statistical analyses

The study was undertaken with the aim of measuring two-dose protection over 3 years of follow-up. There is a paucity of data for vaccine effectiveness (VE) of Euvichol-Plus. Because of a sufficient number of cholera cases that were recruited in 1 year, we decided to carry out an interim analysis of VE after 1 year of follow-up (21 August 2022 to 20 August 2023). For the primary interim analysis, the targeted exposure was the receipt of two doses of Euvichol-Plus at least 14 days before the focal time. Eligible patients with acute watery diarrhoea without visible blood in their stool were included in this analysis. Participants who had received a single dose or who had received two doses but with the second dose less than 14 days before focal time were excluded. The analysis examined VE against medically attended cholera in all age groups by comparing the receipt of two doses vs no doses of the vaccine. Secondary interim analyses considered VE stratified by age at diarrheal symptom onset (1–4 years versus >5 years) and calendar interval (21 August 2022 to 20 February 2023 and 21 February to 20 August 2023) at focal time, as well as against cholera associated with moderate to severe dehydration.

We conducted bivariate analyses to assess the comparability of cases and their matched controls by relevant baseline characteristics, including demographic, behavioural and clinical information, using conditional logistic regression models. To assess the effectiveness of two doses of OCV in matched analyses, we used a conditional logistic regression model with vaccination status as the principal independent variable and matched case-control status as the dependent variable. Variables associated with case-control status at p<0.10 in the bivariate analysis were introduced as independent variables in the models (table 1 and onlinesupplemental tables 1^2^, onlinesupplemental tables 3^4^, onlinesupplemental tables 5^6^, onlinesupplemental tables 7^8^).

**Table 1:** Baseline characteristics of culture-confirmed cholera cases and matched controls

Characteristics	Cases, n=226 (%)	Matched controls, n=552 (%)	P value
Age (years)	25.3±16.2*	28.8±18.4*	0.002
Age groups			
1–4 years	40 (17.7)	103 (18.7)	0.256
5–17 years	25 (11.1)	42 (7.6)	
18–59 years	155 (68.6)	381 (69.0)	
≥60 years	6 (2.7)	26 (4.7)	
Gender (male)	114 (50.4)	291 (52.7)	0.703
Household monthly expenditure (Bangladeshi Taka)†	14 721±8087*	18 640±16 013*	<0.001
Shared toilet	144 (63.7)	291 (52.7)	0.025
Shared kitchen	147 (65)	300 (54.3)	0.035
Safe source of drinking water	45 (19.9)	126 (22.8)	0.208
Treated drinking water	151 (66.8)	386 (69.9)	0.531
Underground water tank	111 (49.1)	294 (53.3)	0.417
Disinfectant used in underground water tank	67 (60.4)	171 (58.2)	0.177
Handwashing after defecation	212 (93.8)	525 (95.1)	0.581
Handwashing before eating	198 (87.6)	501 (90.8)	0.216

* Mean±standard deviationSD.

† Conversion rate: 1 USD=103 Bangladeshi Taka.

The OR for the relationship between vaccination and disease status was estimated by exponentiating the vaccination coefficient from the fitted model. OCV effectiveness was calculated as [(1−OR) × 100]. To enable the primary comparison to be assessed at p<0.05 in the final analysis (after 3 years of follow-up), we followed the O'Brien-Fleming strategy for p value spending in our interim analysis.^15^ Accordingly, we considered p<0.005 (2-tailed) as the threshold of significance for the primary comparison in this interim analysis, taking p<0.05 (2-tailed) as the threshold for secondary analyses. Using this p value spending strategy and assuming 50% test-negative controls were vaccinated at the time of presentation, 50% VE among participants of all ages, and considering 80% power, 10% attrition rate (unable to find matched controls), and an average of 2 controls for each case, a minimum of 191 cases and 382 matched controls were required for the primary interim analysis.

To examine the possibility of collider bias,^16^ we also conducted an analysis comparing cases vs controls presenting with moderate to severe dehydration, disregarding the matched selection. For unmatched analyses, we compared cases and controls with the χ² test for categorical variables and the Student’s t-test for dimensional variables and used unconditional logistic regression models to estimate VE, using the same criteria as for the conditional models for introducing baseline variables as covariates. In addition, in the unconditional regression models, the variables used for matching cases and controls (ie, age group at diarrheal onset, calendar intervals [21 August 2022 to 20 February 2023 and 21 February to 20 August 2023], and study site) were forced into the models as independent variables.

All analyses were performed after a formal data lock and according to analyses outlined in a statistical analysis plan. We used R statistical software (version 4.10) for data analysis. The Clogit package of R was used to estimate the conditional logistic regression coefficients by maximising the conditional likelihood.

## Results

### Assembly of the study population

**Figure 1:**
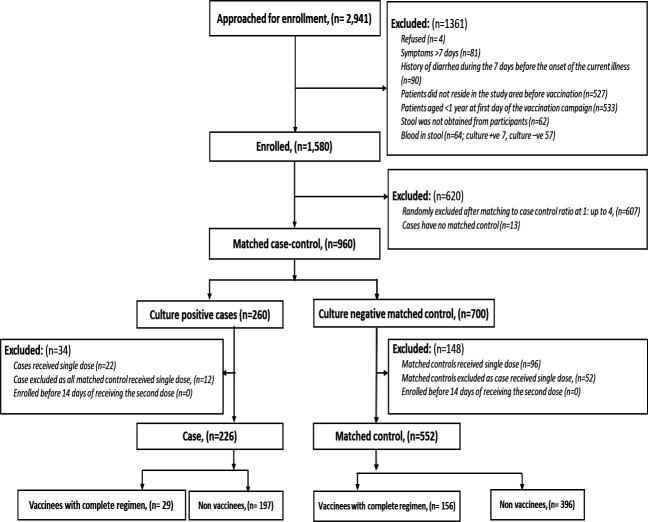
Assembly of the study population.

We approached 2941 patients who presented with acute watery diarrhoea from 21 August 2022 to 20 August 2023, at the two study sites for enrollment. A total of 1580 eligible patients were enrolled after evaluating the eligibility criteria. Among these, we identified 273 (including 48 children aged 1–4 years) culture-confirmed cholera cases (all *V. cholerae* O1 Ogawa) of which 13 were excluded due to a lack of matched controls. From the remaining 260 culture-confirmed cholera cases, 22 were excluded as they received a single dose, and 12 were excluded as their matched controls had received a single dose of vaccine. Finally, a total of 226 cases and 552 matched controls were included in the analysis (figure 1) with most cases and controls identified during the first 6 months of surveillance (online supplemental figure 1).

### Comparability of cases and controls

In the bivariate analysis of matched cases and controls, we found that age, having membership in households with lower expenditure, and households that shared their toilets and kitchens with other households were associated with having culture-confirmed cholera (table 1). A comparison of cholera cases with moderate to severe dehydration and their matched controls showed a significant association with age, household expenditure and hand washing before eating (online supplemental table 1). Online supplemental table 2 shows a simple comparison of cases and controls who were classified as having moderate to severe dehydration at presentation, disregarding the match. Baseline features distinguishing cholera cases from the controls included age, household expenditure, hand washing before eating, calendar intervals, having shared kitchens and using shared toilets.

### Protection associated with the receipt of two doses of Euvichol-plus vaccine

Two-dose receipt of Euvichol-Plus was 13% among cholera cases and 28% among their matched controls (66% VE; 99.5% CI: 30 to 83; p<0.001) (table 2). There was no significant protection (12%; 95% CI: −95 to 60; p=0.756) among children aged 1–4 years, while protection was 79% (95% CI: 60 to 89; p<0.001) for individuals aged ≥5 years. Protection among all age groups was 62% (95% CI: 24 to 81; p=0.006) over the first 6 months and 69% (95% CI: 38 to 85; p=0.001) over the second 6 months of follow-up (table 2). For the analysis of cholera with moderate to severe dehydration using matched controls with any degree of dehydration, protection for all age groups was 69% (95% CI: 44 to 83; p<0.001), while protection among children aged 1–4 years was 6% (95% CI: −206 to 71; p<0.919) and 78% (95% CI: 55 to 89; p<0.001) for individuals ≥5 years of age (table 3). A similar level of protection was observed when the analysis was restricted to cholera cases and test-negative controls who had moderate to severe dehydration: 61% (95% CI: 35 to 78; p=0.001) for all ages; −66% (95% CI: −510 to 54; p<0.436) for children enrolled at 1–4 years and 74% (95% CI: 38 to 91; p<0.001) for persons enrolled at ≥5 years of age (table 4).

**Table 2:** Vaccine effectiveness (VE) of two doses of oral cholera vaccine (OCV) against culture-confirmed cholera stratified by age at focal time and duration of follow-up

Overall period	Cases	Matched controls	Vaccine effectiveness (VE)(95% CI*)			
	Vaccinees n/N (%)	Vaccinees n/N (%)	Crude VE	P value	Adjusted VE	P value
All ages	29/226 (12.8)	156/552 (28.3)	68 (35, 84)	<0.001	66 (30, 83)†‡	<0.001
1–4 years	14/40 (35.0)	39/103 (37.9)	13 (−94, 61)	0.737	12 (−95, 60)§	0.756
≥5 years	15/186 (8.1)	117/449 (26.1)	80 (62, 90)	<0.001	79 (60, 89)†	<0.001
**First 6 months (21 August 2022 to 20 February 2023**)						
All ages	15/136 (11.0)	81/343 (23.6)	65 (31, 82)	0.002	62 (24, 81)¶	0.006
**Second 6 months (21 February to 20 August 2023**)						
All ages	14/90 (15.6)	75/209 (35.9)	71 (41, 86)	0.001	69 (38, 85)**	0.001

* : Confidence intervalAdjusted by age, household monthly expenditure, shared toilet, shared kitchen

† Adjusted by treated drinking water.

‡ 99.5% CI considered for the PE calculation.

§ Adjusted by treated drinking water

¶ Adjusted by age, household monthly expenditure, shared toilet and shared kitchen

** Adjusted by age.

**Table 3:** Vaccine effectiveness (VE) of two doses of oral cholera vaccine (OCV) against culture-confirmed cholera with moderate to severe dehydration stratified by age at focal time, 21 August 2022 to 20 August 2023

Ages	Cases	Matched controls	Vaccine effectiveness (VE) (95% CI*)			
	Vaccinees n/N (%)	Vaccinees n/N (%)	Crude VE	P value	Adjusted VE	P value
All ages	20/184 (10.9)	119/449 (26.5)	72 (49, 84)	<0.001	69 (44, 83)†	<0.001
1–4 years	8/21 (38.1)	20/50 (40.0)	16 (−164, 73)	0.768	6 (−206, 71)‡	0.919
≥5 years	12/163 (7.4)	99/399 (24.8)	79 (58, 89)	<0.001	78 (55, 89)§	<0.001

* CI: Confidence interval.

† Adjusted by age, HH expenditure, shared toilet, and hand washing before eating.

‡ Adjusted by hand washing before eating.

§ Adjusted by HH expenditure, and shared toilet.

**Table 4:** Vaccine effectiveness (VE) of two doses of oral cholera vaccine (OCV) against culture-confirmed cholera with moderate to severe dehydration, using unmatched controls with moderate to severe dehydration, stratified by age at focal time, 21 August 2022 to 20 August 2023

Ages	Cases	Controls	Vaccine effectiveness (VE)(95% CI*)			
	Vaccinees n/N (%)	Vaccinees n/N (%)	Crude VE	P value	Adjusted VE	P value
All ages	20/184 (10.9)	99/404 (24.5)	62 (38, 78)	<0.001	61 (35, 78)†	0.001
1–4 years	8/21 (38.1)	11/38 (28.9)	−51 (−368, 52)	0.473	−66 (−510, 54)‡	0.436
≥5 years	12/163 (7.4)	88/366 (24.0)	75 (54, 87)	<0.001	74 (38, 91)§	<0.001

* CI: Confidence interval.

† Adjusted by age, study site, duration (first 6 months), HH expenditure, shared toilet, shared kitchen, and hand washing before eating.

‡ Adjusted by study site, and duration (first 6 months).

§ Adjusted by study site, duration (first 6 months), and HH expenditure.

## Discussion

We found that a two-dose regimen of the OCV, Euvichol-Plus, delivered to control a major outbreak of cholera in Dhaka in 2022, was associated with 66% protection against cholera overall in the first year after vaccination but no evidence of protection in children 1–4 years of age in this interim analysis. Similar levels of protection were observed against cholera associated with moderate or severe dehydration. No decline in protection against cholera regardless of severity was observed between 0–6 and 6–12 months after vaccination. Notably, previous studies have reported that the duration of protection or the robustness of the immune responses following natural infection with cholera remains unclear.^17^ Additionally, it has been shown that natural infections resulting in more severe diseases lead to stronger long-term protection.^18 19^ Other evidence indicates that subclinical infections result in less robust protection than clinical infections.^17^

Potential limitations of the study require consideration. Our study was observational and did not employ the safeguards of randomisation and double-blinding that help ensure the validity of randomised, double-blinded trials. Additionally, the window for enrollment was limited to the hours of 8:30–17:00 when adults may be at work and children at school; thus, eligible patients presenting outside that window were not approached for enrollment. This may lead to a selection bias of those children who had the ability to leave school and adults able to leave work to seek care during these hours. Although our study was adequately powered for 1 year VE for all age groups, the power was limited for children <5 years of age. Despite this, our study had several strengths. The use of the sampling frame provided by the test-negative design allowed the selection of controls who could have been selected as cases, a key assumption of case-control studies, and helped minimise bias due to differential health-seeking behaviour for the care of diarrhoea between vaccinees and non-vaccinees. The validity of the study was further enhanced by adjusting for potential confounding variables, prospective conduct of the study, determination of eligibility and acquisition of informed consent without knowledge of the culture results of cases and controls, as well as the ascertainment of vaccination status based on objectively verified sources, obtained in a fashion without knowledge of the participants’ status as cases or controls. Also, the study population was assembled using incidence sampling of subjects who were present at baseline, which helped guard against biases that can be introduced by the post-vaccination migration of the population. Finally, as health facilities were used as a sampling frame for the selection of cases and controls, estimates of protection might be distorted by collider bias. However, similar findings for VE were observed when the analysis was restricted to patients with moderate to severe dehydration, in whom healthcare-seeking behaviour was likely non-discretionary.

The level of VE associated with a two-dose regimen of Euvichol-Plus in this study among all vaccinated age groups was similar to levels observed in past evaluations of two-dose regimens of Shanchol and Euvichol, vaccines identical in composition to Euvichol-Plus, in populations comprising both children and adults. A cluster randomised trial of Shanchol carried out in Dhaka, Bangladesh, revealed a moderate level of protection (53%) with two doses against severe cholera during 2 years of follow-up.^20^ Similarly, an earlier randomised trial of Shanchol carried out in Kolkata, India, showed 65% cumulative protection for two doses against all episodes of cholera during 5 years of follow-up.^21^ Observational studies of Shanchol carried out in Haiti, India and Guinea reported 62%, 65% and 87% protection against all episodes of cholera, respectively.^22^,^24^ Another case-control study with Euvichol conducted in Haiti revealed 69% protection after receipt of two doses but was limited by a wide CI due to a very small number of cases.^25^ A somewhat higher level of VE (81%) against cholera was observed for age groups 1 year and older vaccinated with two doses of Euvichol-Plus in Zambia, and a case-control study in the DRC revealed 52.7% and 44.7% protection with a single dose of the vaccine during 12–17 months and 24–36 months after vaccination, respectively.^8 9^

Previous studies of OCVs with the same composition as Euvichol-Plus but with a different presentation (ie, glass vials vs plastic squeeze tubes) reported point estimates corresponding to moderate levels of protection against cholera in children 1–4 years of age. The protection conferred by two doses of the OCV Shanchol was 42% (95% CI: 5 to 64) for 5 years and 50% (95% CI: −850 to 97) for 2 years in India and Haiti, respectively.^21 24^ Similarly, a study of Shanchol in Bangladesh showed 44% (95% CI: −35 to 77) protection against severe cholera among children aged 1–4 years.^20^ However, our study failed to demonstrate or suggest OCV protection in children 1–4 years of age. Similarly, a recent study of Euvichol-Plus conducted in Zambia revealed no protection (−23%; 95% CI: −1013 to 86) against cholera among children under 5 years old who were given at least one dose of a two-dose regimen (estimate calculated from the authors’ data).^8^ In contrast, a recent study of a single dose of the Euvichol-Plus conducted in the DRC demonstrated 73.5% (95% CI: 28.9 to 90.1) vaccine effectiveness for children aged 1–4 years during 12–17 months after vaccination which declined 32.9% (95% CI: −30.7 to 65.5) within 24–36 months after vaccination. However, in contrast to our study, the DRC study lacked objective corroboration of vaccination histories in a substantial proportion of participants.^9^ Previous studies have described potential reasons for differences in immune responses between children and adults following infection and vaccination, including genetic polymorphisms, nutrition, micronutrient status, blood group and coinfection.^26^

While the overall level of VE in this study is reassuring, the absence of VE in young children in this study as well as in an earlier study of Euvichol-Plus in Zambia is potentially concerning.^8^ The 95% CIs surrounding estimates of protection among young children in our study and the Zambian study cannot rule out statistical variation as an explanation for our findings.^8^ This indicates the need for further investigation of Euvichol-Plus vaccine effectiveness among young children. However, if real, our observations could reflect the different presentation of this vaccine, entailing single-dose flexible tubes that may not guarantee complete delivery of the inactivated cholera whole-cell contents, a deficiency that might particularly affect the protection of young children, who in contrast to adults are not immunologically primed before vaccination in cholera-endemic populations. Our findings therefore constitute a signal, requiring further evaluation in future studies.

Additionally, we are continuing surveillance and planning to assess the durability of protection after 3 years of follow-up. A meta-analysis by Bi *et al* reported that the efficacy of killed OCV decreased to 39% by the third year.^27^ Therefore, the final analysis, which will build on this interim analysis, will provide insights into the trend of decreasing protection over time. This could serve as a benchmark for evaluating the long-term sustainability of protection provided by Euvichol-Plus in this cohort.

## supplementary material

10.1136/bmjgh-2024-016571 online supplemental figure 1

10.1136/bmjgh-2024-016571 online supplemental table 1

10.1136/bmjgh-2024-016571 online supplemental table 2

10.1136/bmjgh-2024-016571 online supplemental table 3

10.1136/bmjgh-2024-016571 online supplemental table 4

10.1136/bmjgh-2024-016571 online supplemental table 5

10.1136/bmjgh-2024-016571 online supplemental table 6

10.1136/bmjgh-2024-016571 online supplemental table 7

10.1136/bmjgh-2024-016571 online supplemental table 8

## Data Availability

No data are available.

## References

[1] Dan-Nwafor CC, Ogbonna U, Onyiah P (2019). A cholera outbreak in a rural north central Nigerian community: an unmatched case-control study. BMC Public Health.

[2] WHO (2023). Cholera.

[3] Pezzoli L, Oral Cholera Vaccine Working Group of the Global Task Force on Cholera Control (2020). Global oral cholera vaccine use, 2013-2018. Vaccine (Auckl).

[4] Ali M, Qadri F, Kim DR (2021). Effectiveness of a killed whole-cell oral cholera vaccine in Bangladesh: further follow-up of a cluster-randomised trial. Lancet Infect Dis.

[5] Shah S, Nandy RK, Sethi SS (2023). Comparison of the immunogenicity and safety of Euvichol-Plus with Shanchol in healthy Indian adults and children: an open-label, randomised, multicentre, non-inferiority, parallel-group, phase 3 trial. *Lancet Reg Health Southeast Asia*.

[6] Odevall L, Hong D, Digilio L (2018). The Euvichol story - Development and licensure of a safe, effective and affordable oral cholera vaccine through global public private partnerships. Vaccine (Auckl).

[7] (2023). IVI.

[8] Sialubanje C, Kapina M, Chewe O (2022). Effectiveness of two doses of Euvichol-plus oral cholera vaccine in response to the 2017/2018 outbreak: a matched case-control study in Lusaka, Zambia. BMJ Open.

[9] Malembaka EB, Bugeme PM, Hutchins C (2024). Effectiveness of one dose of killed oral cholera vaccine in an endemic community in the Democratic Republic of the Congo: a matched case-control study. Lancet Infect Dis.

[10] Khan ZH, Islam MT, Amin MA (2024). The reactive cholera vaccination campaign in urban Dhaka in 2022: experience, lessons learned and future directions. *Public Health Pract (Oxf*).

[11] WHO Guidelines Approved by the Guidelines Review Committee (2013). Pocket Book of Hospital Care for Children: Guidelines for the Management of Common Childhood Illnesses.

[12] WHO (2014). Integrated management of childhood illness - chart booklet (march 2014).

[13] Rennels MB, Levine MM, Daya V (1980). Selective vs. nonselective media and direct plating vs. enrichment technique in isolation of Vibrio cholerae: recommendations for clinical laboratories. J Infect Dis.

[14] Kim D, Hong J, Choi Y (2020). Generation and Characterization of Monoclonal Antibodies to the Ogawa Lipopolysaccharide of *Vibrio cholerae* O1 from Phage-Displayed Human Synthetic Fab Library. J Microbiol Biotechnol.

[15] Fleming TR, Harrington DP, O’Brien PC (1984). Designs for group sequential tests. Control Clin Trials.

[16] Tönnies T, Kahl S, Kuss O (2022). Collider Bias in Observational Studies. Dtsch Arztebl Int.

[17] Leung T, Matrajt L Immune responses to cholera following natural infection: a review. Infectious Diseases (except HIV/AIDS).

[18] Clements ML, Levine MM, Young CR (1982). Magnitude, kinetics, and duration of vibriocidal antibody responses in North Americans after ingestion of Vibrio cholerae. J Infect Dis.

[19] Kaper JB, Morris JG, Levine MM (1995). Cholera. Clin Microbiol Rev.

[20] Qadri F, Ali M, Chowdhury F (2015). Feasibility and effectiveness of oral cholera vaccine in an urban endemic setting in Bangladesh: a cluster randomised open-label trial. Lancet.

[21] Bhattacharya SK, Sur D, Ali M (2013). 5 year efficacy of a bivalent killed whole-cell oral cholera vaccine in Kolkata, India: a cluster-randomised, double-blind, placebo-controlled trial. Lancet Infect Dis.

[22] Luquero FJ, Grout L, Ciglenecki I (2014). Use of Vibrio cholerae vaccine in an outbreak in Guinea. N Engl J Med.

[23] Sur D, Kanungo S, Sah B (2011). Efficacy of a low-cost, inactivated whole-cell oral cholera vaccine: results from 3 years of follow-up of a randomized, controlled trial. PLoS Negl Trop Dis.

[24] Ivers LC, Hilaire IJ, Teng JE (2015). Effectiveness of reactive oral cholera vaccination in rural Haiti: a case-control study and bias-indicator analysis. Lancet Glob Health.

[25] Matias WR, Guillaume Y, Cene Augustin G (2024). Effectiveness of the Euvichol® oral cholera vaccine at 2 years: A case-control and bias-indicator study in Haiti. Int J Infect Dis.

[26] Levine MM (2010). Immunogenicity and efficacy of oral vaccines in developing countries: lessons from a live cholera vaccine. BMC Biol.

[27] Bi Q, Ferreras E, Pezzoli L (2017). Protection against cholera from killed whole-cell oral cholera vaccines: a systematic review and meta-analysis. Lancet Infect Dis.

