# Supplementary figures and images for: Evaluation of oral cholera vaccine (Euvichol-Plus) effectiveness against Vibrio cholerae in Bangladesh: an interim analysis
Source: BMJ Glob Health. 2025 Feb 3;10(2):e016571. doi: 10.1136/bmjgh-2024-016571 (PMC11795403; doi:10.1136/bmjgh-2024-016571)

**Figure 2: Numbers of patients enrolled during the study period (August 21, 2022-August 20, 2023)**

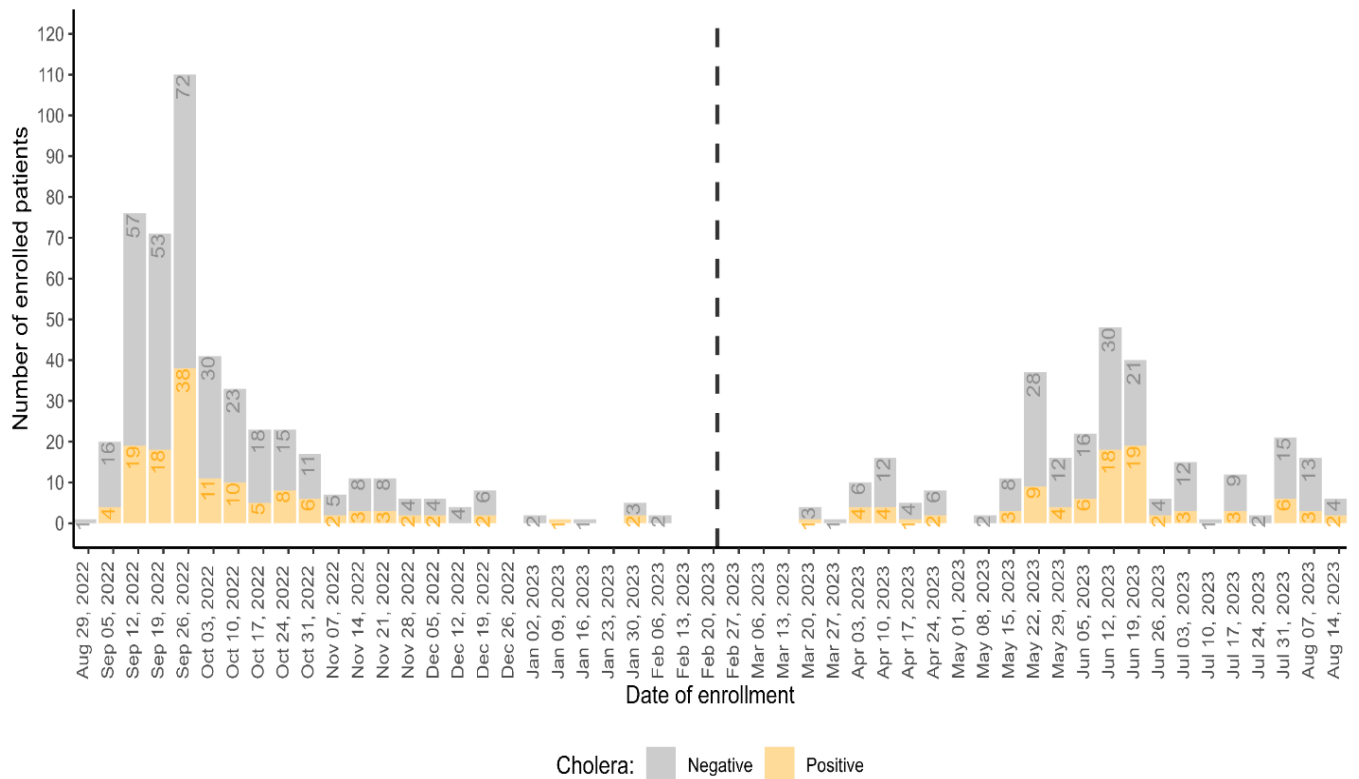

Supplement: online supplemental figure 1 [file bmjgh-10-2-s001.pdf]
