# Supplementary material for: Evaluation of oral cholera vaccine (Euvichol-Plus) effectiveness against Vibrio cholerae in Bangladesh: an interim analysis
Source: BMJ Glob Health. 2025 Feb 3;10(2):e016571. doi: 10.1136/bmjgh-2024-016571 (PMC11795403; doi:10.1136/bmjgh-2024-016571)
Supplement: online supplemental table 1 [file bmjgh-10-2-s002.pdf]

Supplementary Table 1. Baseline characteristics of culture-confirmed cholera cases with moderate to severe dehydration and their matched controls

**Characteristics**	**Cases, n=184 (%)**	**Matched controls, n=459 (%)**	***P* value**
Age (years)	27.3±15.5*	31.4±17.3*	0.004
Age groups			
1–4 years	21 (11.4)	50 (11.1)	0.226
5–17 years	21 (11.4)	37 (8.2)	
18–59 years	137 (74.5)	338 (75.3)	
≥60 years	5 (2.7)	24 (5.3)	
Gender (male)	89 (48.4)	234 (52.1)	0.428
Household monthly expenditure (Bangladeshi Taka)†	14413±7391*	18280±16637*	<0.001
Shared toilet	123 (66.8)	253 (56.3)	0.059
Shared kitchen	124 (67.4)	258 (57.5)	0.102
Safe source of drinking water	41 (22.3)	104 (23.2)	0.621
Treated drinking water	117 (63.6)	309 (68.8)	0.356
Underground water tank	87 (47.3)	229 (51.0)	0.536
Disinfectant underground water tank	52 (59.8)	139 (60.7)	0.209
Hand washing after defecation	171 (92.9)	429 (95.5)	0.292
Hand washing before eating	158 (85.9)	411 (91.5)	0.050

*Mean±standard deviation

†Conversion rate: 1USD=103 Bangladeshi Taka
