# Supplementary material for: Evaluation of oral cholera vaccine (Euvichol-Plus) effectiveness against Vibrio cholerae in Bangladesh: an interim analysis
Source: BMJ Glob Health. 2025 Feb 3;10(2):e016571. doi: 10.1136/bmjgh-2024-016571 (PMC11795403; doi:10.1136/bmjgh-2024-016571)
Supplement: online supplemental table 2 [file bmjgh-10-2-s003.pdf]

**Supplementary Table 2. Baseline characteristics of culture-confirmed cholera cases and controls with moderate to severe dehydration**

| Characteristics | Cases, n=184 (%) | Controls, n=404 (%) | P value |
|---|---|---|---|
| Study site (Dakshinkhan) | 62(33.7) | 147(36.4) | 0.528 |
| Calendar intervals (August 21, 2022 to February 20, 2023) | 108 (58.7) | 236 (58.4) | 0.949 |
| Age (years) | 27.3 ± 15.5* | 32.0 ± 16.9* | 0.001 |
| Age groups | | | |
| 1–4 years | 21(11.4) | 38(9.4) | 0.766 |
| 5–17 years | 21(11.4) | 34(8.4) | |
| 18–59 years | 137(74.5) | 309(76.5) | |
| ≥60 years | 5(2.7) | 23(5.7) | |
| Gender (male) | 89(48.4) | 198(49.0) | 0.886 |
| Household monthly expenditure (Bangladeshi Taka)† | 14413 ± 7392* | 18235 ± 16063* | 0.002 |
| Shared toilet | 123(66.8) | 231(57.2) | 0.026 |
| Shared kitchen | 124(67.4) | 236(58.4) | 0.038 |
| Safe source of drinking water | 41(22.3) | 94(23.3) | 0.793 |
| Treated drinking water | 117(63.6) | 274(67.8) | 0.314 |
| Underground water tank | 87(47.3) | 201(49.8) | 0.579 |
| Disinfectant underground water tank | 52(59.8) | 117(58.2) | 0.806 |
| Hand washing after defecation | 171(92.9) | 385(95.3) | 0.242 |
| Hand washing before eating | 158(85.9) | 368(91.1) | 0.056 |

*Mean±standard deviation

†Conversion rate: 1USD=103 Bangladeshi Taka
