# Supplementary material for: Evaluation of oral cholera vaccine (Euvichol-Plus) effectiveness against Vibrio cholerae in Bangladesh: an interim analysis
Source: BMJ Glob Health. 2025 Feb 3;10(2):e016571. doi: 10.1136/bmjgh-2024-016571 (PMC11795403; doi:10.1136/bmjgh-2024-016571)
Supplement: online supplemental table 3 [file bmjgh-10-2-s004.pdf]

Supplementary Table 3. Baseline characteristics of culture-confirmed cholera cases and matched controls in individuals aged <5 years

Characteristics	Cases, n=40 (%)	Controls, n=103 (%)	p-value
Age (years)	2.4 ± 1.1*	1.8 ± 1*	0.009
Gender (male)	24(60)	65(63.1)	0.862
Household monthly expenditure (Bangladeshi Taka) †	17525 ± 10389.8	18844.7 ± 8235.2	0.465
Shared toilet	18(45)	34(33)	0.298
Shared kitchen	20(50)	37(35.9)	0.239
Safe source of drinking water	4(10)	19(18.4)	0.154
Treated drinking water	37(92.5)	78(75.7)	0.047
Underground water tank	22(55)	67(65)	0.393
Disinfectant underground water tank	15(68.2)	32(47.8)	0.946
Hand washing after defecation	37(92.5)	96(93.2)	0.627
Hand washing before eating	35(87.5)	95(92.2)	0.314

*Mean±standard deviation

†Conversion rate: 1USD=103 Bangladeshi Taka
