# Supplementary material for: Evaluation of oral cholera vaccine (Euvichol-Plus) effectiveness against Vibrio cholerae in Bangladesh: an interim analysis
Source: BMJ Glob Health. 2025 Feb 3;10(2):e016571. doi: 10.1136/bmjgh-2024-016571 (PMC11795403; doi:10.1136/bmjgh-2024-016571)
Supplement: online supplemental table 4 [file bmjgh-10-2-s005.pdf]

Supplementary Table 4. Baseline characteristics of culture-confirmed cholera cases and matched controls in individuals aged ≥5 years

Characteristics	Cases, n=186 (%)	Controls, n=449 (%)	p-value
Age (years)	30.3 ± 13.4*	35 ± 14.5*	0.001
Gender (male)	90(48.4)	226(50.3)	0.621
Household monthly expenditure (Bangladeshi Taka) †	14408.6 ± 6838.3	17787.3 ± 10054.6	0
Shared toilet	126(67.7)	257(57.2)	0.047
Shared kitchen	127(68.3)	263(58.6)	0.077
Safe source of drinking water	41(22)	107(23.8)	0.487
Treated drinking water	114(61.3)	308(68.6)	0.125
Underground water tank	89(47.8)	227(50.6)	0.619
Disinfectant underground water tank	52(58.4)	139(61.2)	0.102
Hand washing after defecation	175(94.1)	429(95.5)	0.709
Hand washing before eating	163(87.6)	406(90.4)	0.366

*Mean±standard deviation

†Conversion rate: 1USD=103 Bangladeshi Taka
