# Supplementary material for: Evaluation of oral cholera vaccine (Euvichol-Plus) effectiveness against Vibrio cholerae in Bangladesh: an interim analysis
Source: BMJ Glob Health. 2025 Feb 3;10(2):e016571. doi: 10.1136/bmjgh-2024-016571 (PMC11795403; doi:10.1136/bmjgh-2024-016571)
Supplement: online supplemental table 5 [file bmjgh-10-2-s006.pdf]

Supplementary Table 5. Baseline characteristics of culture-confirmed cholera cases with moderate to severe dehydration and their matched controls in individuals aged <5 years

Characteristics	Cases, n=21(%)	Controls, n=50(%)	p-value
Age (years)	2.4 ± 1.1*	1.9 ± 1*	0.046
Gender (male)	13(61.9)	32(64)	0.783
Household monthly expenditure (Bangladeshi Taka) †	17095.2 ± 8055.5	18460 ± 7656.4	0.481
Shared toilet	11(52.4)	20(40)	0.42
Shared kitchen	11(52.4)	21(42)	0.598
Safe source of drinking water	4(19)	6(12)	0.74
Treated drinking water	20(95.2)	40(80)	0.139
Underground water tank	11(52.4)	30(60)	0.786
Disinfectant underground water tank	6(54.5)	15(50)	0.564
Hand washing after defecation	19(90.5)	48(96)	0.288
Hand washing before eating	17(81)	48(96)	0.065

*Mean±standard deviation

†Conversion rate: 1USD=103 Bangladeshi Taka
