# Supplementary material for: Evaluation of oral cholera vaccine (Euvichol-Plus) effectiveness against Vibrio cholerae in Bangladesh: an interim analysis
Source: BMJ Glob Health. 2025 Feb 3;10(2):e016571. doi: 10.1136/bmjgh-2024-016571 (PMC11795403; doi:10.1136/bmjgh-2024-016571)
Supplement: online supplemental table 6 [file bmjgh-10-2-s007.pdf]

Supplementary Table 6. Baseline characteristics of culture-confirmed cholera cases with moderate to severe dehydration and their matched controls in individuals aged ≥5 years

Characteristics	Cases, n=163(%)	Controls, n=399(%)	p-value
Age (years)	30.5 ± 13.4*	35.1 ± 14.6*	0.003
Gender (male)	76(46.6)	202(50.6)	0.363
Household monthly expenditure (Bangladeshi Taka) †	14220.9 ± 6569.7	17324.6 ± 8480.7	0
Shared toilet	112(68.7)	233(58.4)	0.087
Shared kitchen	113(69.3)	237(59.4)	0.121
Safe source of drinking water	37(22.7)	98(24.6)	0.518
Treated drinking water	97(59.5)	269(67.4)	0.152
Underground water tank	76(46.6)	199(49.9)	0.576
Disinfectant underground water tank	46(60.5)	124(62.3)	0.258
Hand washing after defecation	152(93.3)	381(95.5)	0.467
Hand washing before eating	141(86.5)	363(91)	0.164

*Mean±standard deviation

†Conversion rate: 1USD=103 Bangladeshi Taka

``
