# Supplementary material for: Evaluation of oral cholera vaccine (Euvichol-Plus) effectiveness against Vibrio cholerae in Bangladesh: an interim analysis
Source: BMJ Glob Health. 2025 Feb 3;10(2):e016571. doi: 10.1136/bmjgh-2024-016571 (PMC11795403; doi:10.1136/bmjgh-2024-016571)
Supplement: online supplemental table 7 [file bmjgh-10-2-s008.pdf]

Supplementary Table 7. Baseline characteristics of culture-confirmed cholera cases and controls with moderate to severe dehydration in individuals aged <5 years

**Characteristics**	**Cases, n=163(%)**	**Controls, n=399(%)**	**p-value**
Age (years)	30.5 ± 13.4*	35.1 ± 14.6*	0.003
Gender (male)	76(46.6)	202(50.6)	0.363
Household monthly expenditure (Bangladeshi Taka) †	14220.9 ± 6569.7	17324.6 ± 8480.7	0
Shared toilet	112(68.7)	233(58.4)	0.087
Shared kitchen	113(69.3)	237(59.4)	0.121
Safe source of drinking water	37(22.7)	98(24.6)	0.518
Treated drinking water	97(59.5)	269(67.4)	0.152
Underground water tank	76(46.6)	199(49.9)	0.576
Disinfectant underground water tank	46(60.5)	124(62.3)	0.258
Hand washing after defecation	152(93.3)	381(95.5)	0.467
Hand washing before eating	141(86.5)	363(91)	0.164

**Characteristics**	**Cases, n=21(%)**	**Controls, n=38(%)**	**p-value**
Age (years)	2.4 ± 1.1*	2 ± 1.1*	0.134
Gender (male)	13(61.9)	28(73.7)	0.297
Household monthly expenditure (Bangladeshi Taka) †	17095.2 ± 8055.5	19578.9 ± 8842.9	0.358
Shared toilet	11(52.4)	16(42.1)	0.629
Shared kitchen	11(52.4)	17(44.7)	0.909
Safe source of drinking water	4(19)	8(21.1)	0.524
Treated drinking water	20(95.2)	27(71.1)	0.306
Underground water tank	11(52.4)	22(57.9)	0.669
Disinfectant underground water tank	6(54.5)	8(36.4)	0.488
Hand washing after defecation	19(90.5)	37(97.4)	0.624
Hand washing before eating	17(81)	37(97.4)	0.624

*Mean±standard deviation

†Conversion rate: 1USD=103 Bangladeshi Taka
