# Supplementary material for: Evaluation of oral cholera vaccine (Euvichol-Plus) effectiveness against Vibrio cholerae in Bangladesh: an interim analysis
Source: BMJ Glob Health. 2025 Feb 3;10(2):e016571. doi: 10.1136/bmjgh-2024-016571 (PMC11795403; doi:10.1136/bmjgh-2024-016571)
Supplement: online supplemental table 8 [file bmjgh-10-2-s009.pdf]

Supplementary Table 8. Baseline characteristics of culture-confirmed cholera cases and controls with moderate to severe dehydration in individuals aged ≥5 years

**Characteristics**	Cases, n=163(%)	Controls, n=366(%)	p-value
Age (years)	30.5 ± 13.4*	35.1 ± 14.5*	0.003
Gender (male)	76(46.6)	170(46.4)	0.997
Household monthly expenditure (Bangladeshi Taka) †	14220.9 ± 6569.7	17431.7 ± 9348	0
Shared toilet	112(68.7)	215(58.7)	0.131
Shared kitchen	113(69.3)	219(59.8)	0.204
Safe source of drinking water	37(22.7)	86(23.5)	0.346
Treated drinking water	97(59.5)	247(67.5)	0.424
Underground water tank	76(46.6)	179(48.9)	0.987
Disinfectant underground water tank	46(60.5)	109(60.9)	0.283
Hand washing after defecation	152(93.3)	348(95.1)	0.507
Hand washing before eating	141(86.5)	331(90.4)	0.162

*Mean±standard deviation

†Conversion rate: 1USD=103 Bangladeshi Taka
